# Moving from table to graph in physics-informed spatio-temporal symbolic regression

**DOI:** 10.1038/s41598-026-53882-w

**Published:** 2026-05-23

**Authors:** Teddy Lazebnik, Alex Liberzon

**Affiliations:** 1 Department of Information Systems, University of Haifa, Haifa, Israel; 2 Department of Computing, Jonkoping University, Jonkoping, Sweden; 3 Turbulence Structure Laboratory, School of Mechanical Engineering, Tel Aviv University, Tel Aviv, Israel

**Keywords:** Graph neural networks, Equation discovery, Implicit knowledge-integration, Scientific machine learning, Mathematics and computing, Physics

## Abstract

Symbolic Regression (SR) is a powerful technique for discovering analytical mathematical expressions that describe observed numerical data. Traditionally, SR models work on data in tabular form, imposing a purely functional mapping without considering the underlying spatio-temporal dependencies or the governing physical laws. Such approaches are ill-suited for physical problems, where data evolves dynamically across time and space and is governed by ordinary/partial differential equations (ODEs/PDEs). Moreover, as SR are commonly measured by their ability to obtain generalized equations from a relatively small amount of data, representing the data efficiently plays a central role in the performance of such models. In this study, we propose a simple yet powerful solver-agnostic approach for SR fitting by using a dual representation — one that preserves explainability, while the other is physically informed. Our main novelty lies in combining the standard tabular representation with a graph-based spatio-temporal representation in a unified SR fitting framework that can enhance existing SR solvers without modifying their internal search mechanism. Namely, the method uses both tabular and graph-based data representation, where nodes in the graph are associated with spatio-temporal coordinates and dynamic state variables, while edges encode spatial or temporal dependencies. This approach allows for the direct generation of differential equations that describe the underlying physical system by implicitly incorporating spatio-temporal patterns and constraints. Benchmarks across multiple synthetic datasets originated from functional, ordinary/partial different equations (O/PDE), integral, and delayed ODE, demonstrate the ability of the proposed method to recover governing equations with high accuracy, even in noisy settings, improving a wide range of SR out-of-the box. These results indicate that enriching SR with graph-based spatio-temporal structure provides a practical pathway toward more robust and physically consistent equation discovery. At the same time, the current framework assumes that a meaningful spatio-temporal neighborhood structure can be constructed and is validated primarily on controlled synthetic benchmark systems.

## Introduction

The pursuit of mathematical models that capture the governing principles of natural and engineered systems is a cornerstone of scientific inquiry^1–5^. Across disciplines such as physics^6^, biology^7^, and economics^8^, researchers aim to identify concise and interpretable formulations that describe observed dynamics. To be exact, scientific inquiry, from as early as Isaac Newton, proceeds through a three-step cycle of observation, hypothesis generation, and hypothesis validation^9,10^. Traditionally, researchers begin by collecting data about the world (observation), which they then use to develop explanatory hypotheses (hypothesis generation). A good hypothesis is required to allow for extrapolation and the prediction of new data within the observed system during the hypothesis validation stage^11–13^. Modern science, in general, and modern exact science, in particular, is based on an equation-based formalization of hypothesis^14,15^. This process, more often than not, requires a comprehensive understanding of a field, deep familiarity with the collected observations, and a decent amount of creativity and mathematical expertise^16,17^.

Recently, with the rapid increase in both the volume and complexity of available data, there is a growing need for automated methods that can discover such models directly from observations^18–23^. To this end, scholars developed a wide range of symbolic regression (SR) models, which allow the discovery of analytical expressions that fit given data^24–28^. Unlike black-box machine learning (ML) approaches on the one hand, and simplistic statistical models (such as linear regression), which are explainable but more often than not lacking expressiveness, on the other hand, SR produces interpretable symbolic forms that can be readily analyzed and understood by scientists^29^.

One field in which SR has gained much attention is physics^30–32^. The inherent goal of physics is to uncover governing equations that describe the laws of nature, making SR a natural candidate for accelerating this process^33–35^. Multiple studies have demonstrated the ability of SR to rediscover known physical laws such as Newton’s equations of motion, conservation laws, and simple partial differential equations directly from data^36,37^.

Nonetheless, existing SR models suffer from two limitations that hinder their practical applicability in physics-driven domains. First, SR models assume that data are presented in a tabular form, where each sample is treated independently, without accounting for the spatio-temporal dependencies intrinsic to physical systems. Recent SR models, especially those using Recurrent Neural Network (RNN) based models, partially tackle this limitation as they do consider the temporal context of samples^38–42^. Other works use large language models as generalized and implicit models to support the SR search process^43–45^. In reality, physical processes evolve dynamically in time and space, with local interactions shaping the behavior of the global system. Ignoring these structures leads to models that may fit data but fail to capture the underlying dynamics. Second, SR searches for algebraic expressions mapping variables but does not explicitly account for the governing equations being ordinary or partial differential equations (ODEs/PDEs). Since most physical systems are defined through such equations, neglecting their structure often results in models that lack physical meaning, generalizability, and predictive power.

To this end, in this study, we propose a simple yet powerful data representation approach with SR fitting procedure that aims to tackle these two limitations regardless of the SR method used under the hood. Namely, the proposed method leverages a dual representation of the raw data - one using the traditional tabular representation and the additional graph-based data representation, where nodes encode spatio-temporal states and edges capture spatial or temporal dependencies. This representation allows us to move beyond the traditional tabular paradigm and to directly model the interconnected nature of physical systems. Through a series of benchmarks on synthetic and real-world datasets, we demonstrate that the proposed method is capable of improving the performance of out-of-the-box SR models in recovering governing equations with high fidelity, even under noisy conditions. Hence, the main contributions of this work are:

- We propose a dual tabular–graph representation for spatio-temporal physical data, where nodes represent space–time observations and edges encode spatial/temporal dependencies, enabling SR to leverage physical locality.
- We introduce a solver-agnostic SR fitting objective that combines consistency in the raw feature space with consistency in a physics-aware latent space learned by a GNN, thereby improving equation recovery without modifying the underlying SR solver.
- We provide a comprehensive evaluation across ten SR models and ten physics-derived benchmark systems, including robustness to observational noise and data-efficiency analyses.

**Fig. 1:**
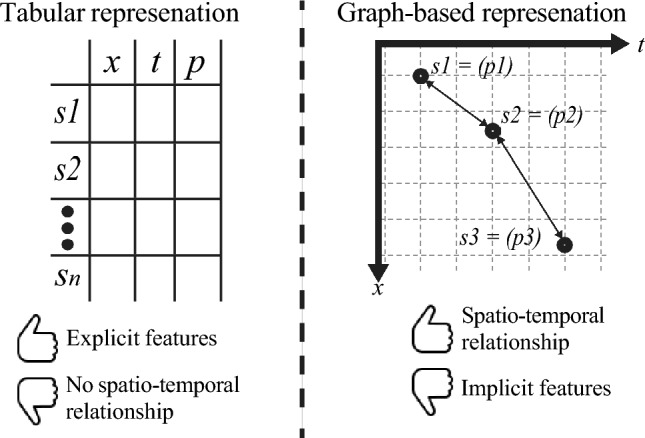
A schematic view of the advantage of the dual tabluar- and graph- based data representation approach.

Figure 1 presents the schematic view of the advantage of our dual-representation approach.

## Related work

SR is a modeling paradigm aimed at uncovering explicit mathematical expressions that capture the relationship between input variables and an output variable^46–48^. Unlike traditional regression approaches such as linear regression, which require a predefined functional form^49^, SR explores a vast space of candidate expressions to identify the one that best fits the observed data^50^. This flexibility makes SR especially valuable in scenarios where the underlying functional form is unknown or highly nonlinear yet required to be expressed via a human-readable equation^51–53^.

Roughly, SR methodologies can be grouped into four categories, depending on the computational strategy employed: brute force, sparse regression, deep learning, and genetic algorithms^54^. Brute-force methods exhaustively enumerate candidate equations, theoretically guaranteeing a solution. However, in practice, they are computationally intractable and prone to severe overfitting^55^. Their main distinction lies in how they partition and traverse the search space^56^. In contrast, sparse regression approaches reduce the search space by exploiting optimization techniques that promote parsimony. A representative example is SINDy, which uses Lasso regression to identify compact models of nonlinear dynamical systems from time series data^57^. SINDy alternates between least-squares fitting and thresholding steps to enforce sparsity^58^. Another family of approaches is based on deep learning. These methods leverage neural networks to generate symbolic expressions, benefiting from robustness against noise and outliers, but often struggle with generalization beyond the fitting domain^59^. For example, the Deep Symbolic Regression (DSR) framework^60^ employs reinforcement learning to train a recurrent neural network (RNN) that generates symbolic formulas, using a risk-seeking policy gradient variant to improve expression discovery. Finally, genetic algorithm–based methods treat candidate equations as individuals in an evolving population^61^. Through selection, crossover, and mutation, the population gradually refines toward expressions that better explain the data. This evolutionary strategy supports interpretability and flexibility, since it does not assume a fixed model class^62,63^. The Python package gplearn, for instance, implements this approach and has been shown to rediscover known physical laws^64^. In gplearn, equations are represented as trees whose substructures evolve via stochastic optimization guided by fitness evaluation. In recent years, several SR toolkits have been proposed, making the methodology increasingly accessible. Widely adopted libraries include DEAP (Distributed Evolutionary Algorithm in Python)^65,66^, gplearn^67,68^, and PySR^69,70^, all of which are based on genetic programming principles.

The application of SR in physics is especially appealing, as scientific domains often involve multivariate, noisy experimental data from nonlinear systems, yet demand interpretable analytical expressions to uncover governing laws^30–32^. However, purely data-driven SR is insufficient in this context, since candidate equations must respect constraints such as dimensional consistency, conservation principles, and symmetries. To address these challenges, Physics-Informed Symbolic Regression (PiSR) has been proposed^32,71^. PiSR integrates prior physical knowledge directly into the search process, guiding model discovery by embedding principles such as conservation laws, scale invariance, or sparsity assumptions^32^.

Several notable approaches exemplify this trend. AI-Feynman combines physics-inspired heuristics with machine learning and deep learning methods, demonstrating strong performance across diverse physics problems^72^. Similarly, the Equation Learner (EQL) framework^73^ uses a specialized feedforward neural network designed to capture dynamical equations and extrapolate beyond observed data. Its architecture integrates linear mappings, nonlinear unary and binary operators, and a linear readout layer, enabling efficient gradient-based fitting. Another contribution is SPRINT (Scalable Pruning for Rapid Identification of Null vecTors), a sparse regression method that generalizes exhaustive search via iterative singular value decomposition (SVD) and pruning, enabling efficient equation discovery without prior model assumptions^74^.

While PISR is tailored to enforce symbolic structure and physical constraints, Physics-Informed Neural Networks (PINNs) represent a parallel and highly active vein of research in scientific machine learning^75–77^. PINNs embed known ordinary/partial differential equations (ODEs/PDEs) into the loss function of neural networks, forcing the learned approximation to satisfy the governing equations (via automatic differentiation) while fitting observed data^78^. Recent reviews highlight that PINNs have become a central pillar in data-driven scientific modeling and inverse problems^79,80^. They often combine boundary/initial condition terms, residual losses from PDE operators, and data mismatch terms into a unified fitting objective^78^.

Recently, several enhancements have been proposed to improve the flexibility, robustness, and interpretability of PINNs. Variants in network architecture, domain decomposition, adaptive collocation sampling, adaptive loss weighting, and hybrid symbolic–neural formulations have all been introduced to mitigate fitting difficulties (e.g. stiffness, imbalance of loss terms) and improve generalization^81^. For example, GPT-PINN frames a meta-learning mechanism, where a meta-network composes pre-trained PINNs as basis units to generalize across parameterized PDEs efficiently^82^. TGPT-PINN extends this to nonlinear model reduction by combining transformed layers and shock-capturing losses^83^. Another line of work, GINN-KAN, seeks to build interpretability into the PINN architecture itself, combining ideas from interpretable networks with classical Kolmogorov–Arnold (KAN) representations so that the resulting PINN is less of a black box^84^. Despite their promising performance, they suffer from key drawbacks. First, fitting is often unstable due to imbalanced loss terms, and scalability to high-dimensional or complex systems is limited by computational cost. Second, PINNs also struggle with sharp features such as shocks or turbulence. Third, they remain largely black-box models without interpretable outputs, and finally, they are sensitive to noisy or sparse boundary conditions. These limitations motivate hybrid approaches with PISR, which can improve interpretability and efficiency while retaining physical consistency.

## Data representation and symbolic regression fitting procedure

The motivation behind the proposed dual representation arises from the fundamental structure of physical reality itself. In physics, systems evolve according to local interactions in space and time — quantities such as velocity, temperature, or concentration at a given point are influenced primarily by their immediate spatial and temporal neighbors. This locality principle underpins the mathematical form of differential equations, where derivatives capture rates of change over infinitesimal distances or times. The tabular representation, while convenient for machine learning, inherently breaks these spatial and temporal linkages by treating each observation as an independent sample. In contrast, a graph representation naturally encodes these dependencies: nodes represent local states, and edges express their physical couplings. By embedding this graph-based structure alongside the traditional tabular view, we hypothesize that the SR process can infer differential relationships more efficiently and therefore, effectively reconstruct the latent operators (e.g., gradients, fluxes, interactions) that govern the system. Thus, the dual representation bridges two complementary perspectives: the table captures measurable data fidelity, while the graph encapsulates physical continuity and causality, together forming a more faithful computational mirror of how nature operates.

**Fig. 2:**
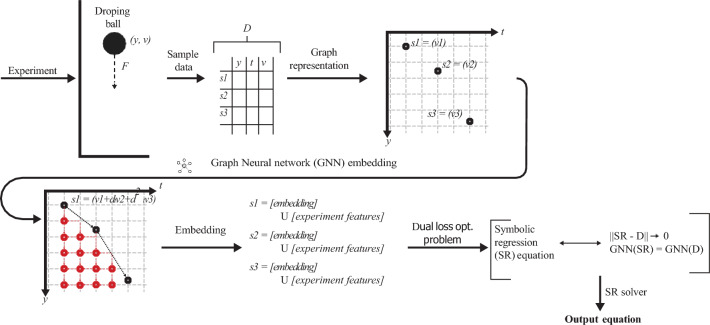
Schematic view of the proposed model.

Formally, the proposed method aims to allow SR models to better discover the underlying governing equations of dynamical systems directly from spatio-temporal data by integrating Graph Neural Networks (GNNs) with SR. The methodology is organized into three principal stages: (i) construction of a spatio-temporal grid graph, (ii) graph-based feature representation learning using GNN, and (iii) SR optimization with a dual representation-based loss function. This architecture enables the recovery of interpretable, physically consistent equations from complex, high-dimensional data. A schematic overview of the $\pi (\boldsymbol{x},t)$SRworkflow is illustrated in Fig. 2.

### Graph representation construction

We assume an experiment in which measurements of one or more physical quantities are collected over time. These quantities may describe the state of a single object (e.g., the position and velocity of a falling ball) or the evolution of a distributed field (e.g., temperature, pressure, or velocity over a fluid domain). In the former case, spatial information may be fixed or limited, whereas in the latter, measurements vary continuously across both space and time. Regardless of the experimental setup, the collected data are reorganized into a unified structure that treats every observation as a node embedded in a four-dimensional space-time manifold ((*x*, *y*, *z*, *t*)), associated with a set of measured features. This graph-based formulation allows the learning model to capture two key forms of dependency:

- Temporal continuity, which ensures that consecutive measurements influence one another in accordance with physical causality.
- Spatial coherence, which enforces locality and smoothness of interactions among neighboring spatial points.

As a result, the learned representation respects the geometry of the physical process and provides a structured substrate upon which SR can operate to infer governing equations. To this end, let the dataset of measurements be denoted by $\mathcal {D} = \{(\textbf{r}_i, t_i, \textbf{f}_i)\}_{i=1}^{N},$ where $\textbf{r}_i = (x_i, y_i, z_i) \in \mathbb {R}^3$ are the spatial coordinates of the *i*-th observation, $t_i \in \mathbb {R}^+$ is the associated time coordinate, and $\textbf{f}_i = [f_{i,1}, f_{i,2}, \dots , f_{i,M}] \in \mathbb {R}^M$ is the vector of measured or derived physical quantities.

From $\mathcal {D}$, we construct an undirected graph $G = (V, \mathbb {E})$, where each node $v_i \in V$ represents a unique space-time observation and is defined as $v_i = \big ((x_i, y_i, z_i, t_i), \textbf{f}_i\big )$ and the edge set $\mathbb {E}$ is composed of spatial and temporal links $\mathbb {E} = \mathbb {E}_S \cup \mathbb {E}_T$ such that spatial edges ($\mathbb {E}_S = \{(v_i, v_j) \mid \Vert \textbf{r}_i - \textbf{r}_j \Vert < \epsilon _S, \ t_i = t_j \}$) connect nodes corresponding to nearby spatial coordinates at the same time step, while temporal edges ($\mathbb {E}_T = \{(v_i, v_j) \mid \textbf{r}_i = \textbf{r}_j, \ |t_i - t_j| < \epsilon _T$) connect nodes corresponding to the same spatial location at consecutive times, where $\epsilon _S$ and $\epsilon _T$ are distance thresholds that determine the radius of spatial and temporal connectivity, respectively. This construction yields a four-dimensional spatio-temporal graph in which the geometry of the physical process is preserved and accessible to graph-based learning models.

Technically, this construction assumes that each observation can be associated with a meaningful spatio-temporal index, either through explicit spatial coordinates and timestamps $(r_i,t_i)$, or through a consistent sensor/mesh indexing that can be mapped to $(r_i,t_i)$. In consequence, the adjacency is derived from a user-defined neighborhood rule (e.g., radius or *k*NN) via the thresholds $\epsilon _S,\epsilon _T$. When the topology changes over time but coordinates remain available (e.g., particle systems or moving sensors), the same pipeline applies by recomputing $\mathbb {E}_S$ and $\mathbb {E}_T$ per time step (or within sliding windows), yielding a dynamic graph sequence. Thus, in such settings, dynamic-graph GNN encoders can be used as drop-in replacements for $\Phi _\Theta$^85,86^. To this end, in problems where spatial relations are unknown or latent, an additional graph-structure-learning component is required to infer the adjacency from data^87^. For example, interaction graphs can be inferred directly from trajectories via latent-relation models^88^.

Importantly, this graph-based structure explicitly defines “local differential relationships” among the components of $\textbf{f}$ in both space and time. For instance, spatial edges approximate partial derivatives $\frac{\partial \textbf{f}}{\partial x}$, $\frac{\partial \textbf{f}}{\partial y}$, and $\frac{\partial \textbf{f}}{\partial z}$ through finite differences between neighboring nodes at constant *t*, while temporal edges approximate temporal derivatives $\frac{\partial \textbf{f}}{\partial t}$ between successive time steps at fixed spatial coordinates. Hence, the graph implicitly encodes the discretized structure of a partial differential operator acting on $\textbf{f}$:

1 $$\begin{aligned} \mathcal {L}(\textbf{f}) \approx \frac{\partial \textbf{f}}{\partial t} + \sum _{k \in \{x, y, z\}} \alpha _k \frac{\partial \textbf{f}}{\partial k} + \beta \nabla ^2 \textbf{f} + \dots , \end{aligned}$$

where $\mathcal {L}$ denotes the latent differential operator governing the physical process, and $\alpha _k, \beta$ are coefficients to be discovered. An example of this phase, where a mass is falling under the influence of gravity, is provided in the Appendix.

### Latent space learning

Once the spatio-temporal graph (*G*) is constructed, it is provided as input to the GNN component. The GNN operates directly on this structured graph representation, transforming node-level physical measurements into a set of learned latent embeddings that encode both spatial and temporal dependencies. These embeddings serve as physics-aware features that the SR module later uses to infer explicit analytical expressions of the underlying dynamics. Technically, each node $v_i \in V$ corresponds to a unique space-time observation $(\textbf{r}_i, t_i)$, with an associated feature vector $\textbf{f}_i \in \mathbb {R}^{M}$ representing the observed or derived physical quantities (e.g., position, velocity, acceleration, temperature, pressure). The initial node embedding is therefore $\textbf{h}_i^{(0)} = \textbf{W}_{\textrm{in}} \textbf{f}_i + \textbf{b}_{\textrm{in}},$ where $\textbf{W}_{\textrm{in}}$ and $\textbf{b}_{\textrm{in}}$ are learnable input projection parameters that map the raw feature space into a latent dimension $d_h$. In addition, each edge $(v_i, v_j)$ carries attributes $e_{ij}$ that may include spatial distance $\Vert \textbf{r}_i - \textbf{r}_j\Vert$, temporal interval $\Delta t = |t_i - t_j|$, or learned positional encoding. In addition, each edge $(v_i, v_j)$ is associated with attributes $e_{ij}$ that may include spatial distance $\Vert \textbf{r}_i - \textbf{r}_j\Vert,$ temporal interval $\Delta t = |t_i - t_j|$, or learned positional encoding. In the general formulation, such attributes may be incorporated into the message function $\psi ^{(l)}$. In our implementation, however, they are used to construct and weight the adjacency matrix, as described in Graph embedding implementation Section.

The GNN updates node embeddings through iterative message-passing operations^89^. At each layer *l*, the node $v_i$ aggregates messages from its neighborhood $\mathcal {N}(i) \subseteq E$ according to:

2 $$\begin{aligned} \textbf{m}_{ij}^{(l)} = \psi ^{(l)}\big (\textbf{h}_i^{(l)}, \textbf{h}_j^{(l)}, e_{ij}\big ), \;\;\;\; \textbf{M}_i^{(l)} = \sum _{j \in \mathcal {N}(i)} \textbf{m}_{ij}^{(l)}, \;\;\; \textbf{h}_i^{(l+1)} = \phi ^{(l)}\big (\textbf{h}_i^{(l)}, \textbf{M}_i^{(l)}\big ), \end{aligned}$$

where $\psi ^{(l)}$ is a message function, $\phi ^{(l)}$ is a node update function, and $\square$ is a permutation-invariant aggregation operator (e.g., sum, mean, or attention-weighted average). In practice, $\psi ^{(l)}$ and $\phi ^{(l)}$ are implemented as multi-layer perceptrons (MLPs) or attention kernels parameterized by $\Theta ^{(l)}$, yielding a differentiable mapping $\mathcal {F}_\Theta : (\textbf{H}^{(l)}, E) \rightarrow \textbf{H}^{(l+1)}$. The formulation above describes a general message-passing framework. In this work, we instantiate it using a Graph Convolutional Network (GCN) where the GCN corresponds to a special case in which messages are aggregated via normalized adjacency weights without explicit edge-conditioned transformations.

Notably, after *L* layers of message passing, each node $v_i$ obtains a latent embedding $\textbf{h}_i^{(L)}$ that encodes not only its instantaneous physical state but also the aggregated influence of its spatio-temporal neighborhood:

$$\textbf{h}_i^{(L)} = \mathcal {F}_\Theta ^{(L)}\!\big (\textbf{f}_i, \{\textbf{f}_j \mid (v_i, v_j) \in \mathbb {E}_S \cup \mathbb {E}_T \}\big ).$$

This embedding space differs fundamentally from the raw measurement space. In the raw data domain, nearby points may appear similar due to sensor noise or limited resolution, obscuring meaningful dynamical variations. In contrast, the GNN-generated latent space emphasizes consistent dynamical relationships: message passing integrates directional information over temporal edges and spatial gradients, effectively filtering out incoherent noise while amplifying physically consistent signals. Consequently, nodes corresponding to regions or time steps governed by the same physical law form coherent clusters in the latent space, while those associated with different regimes or boundary conditions become separable. This property allows the learned representation to be interpreted as a *physics-aware manifold* $\mathcal {M}_\Theta$, where distances reflect similarity in underlying dynamics rather than raw measurements. Within this manifold, trajectories that share identical governing equations follow geometrically smooth paths, whereas perturbations in physical behavior correspond to measurable divergences in latent geometry.

### Symbolic regression fitting procedure

Let us assume *K* independent experiments indexed by $k\in \{1,\dots ,K\}$, each producing a spatio-temporal dataset $\mathcal {D}^{(k)}$ and its graph $G^{(k)}=(V^{(k)},E^{(k)})$. In addition, let $\textbf{F}^{(k)}=\{\textbf{f}_i^{(k)}\}_{i\in V^{(k)}}$ denote the node features (observed/derived physical quantities) on $G^{(k)}$. A trained GNN encoder $\Phi _\Theta$ maps node features on a graph to latent embeddings $\textbf{H}^{(k)} \;=\; \Phi _\Theta \!\big (G^{(k)}, \textbf{F}^{(k)}\big ) \;=\; \{\textbf{h}_i^{(k)}\in \mathbb {R}^{d_h}\}_{i\in V^{(k)}}$.

An SR candidate is an expression $E=(s,\textbf{c})$ with *structure* *s* (an expression tree over a grammar $\mathcal {G}$ of primitives $\{+,-,\times ,\div ,\exp ,\log ,\sin ,\cos ,\nabla ,\nabla ^2,\partial _t,\cdot \}$, etc.) and *constants* $\textbf{c}\in \mathbb {R}^{d_c}$. The expression defines a right-hand side (RHS) operator $\mathcal {R}_{E}$ acting on the state $\textbf{u}$ (scalar or vector field), e.g.,

$$\partial _t \textbf{u} \;=\; \mathcal {R}_{E}\big (\textbf{u},\nabla \textbf{u},\nabla ^2 \textbf{u}, \textbf{x}, t\big ).$$

Given experiment-specific initial/boundary conditions $\mathcal {I}^{(k)}$ and exogenous inputs $\mathcal {U}^{(k)}$, we define a numerical time-stepping simulator $\mathcal {S}_{E}$ (e.g., explicit/implicit Runge–Kutta for ODEs, finite-difference/volume/element for PDEs) that yields a *synthetic* dataset on the same grid/graph:

$$\widehat{\mathcal {D}}^{(k)} \;=\; \mathcal {S}_{E}\big (\mathcal {I}^{(k)},\mathcal {U}^{(k)}\big ), \qquad \widehat{\textbf{F}}^{(k)}=\{\widehat{\textbf{f}}_i^{(k)}\}_{i\in V^{(k)}}, \qquad \widehat{\textbf{H}}^{(k)} \;=\; \Phi _\Theta \!\big (G^{(k)}, \widehat{\textbf{F}}^{(k)}\big ).$$

We assume $\mathcal {S}_E$ produces outputs on the same node set $V^{(k)}$ (or a known interpolation/projection aligns nodes).

The training objective is defined over the set of candidate expressions $E=(s,\textbf{c})$, where each expression is evaluated through the simulator $\mathcal {S}_E$ and the GNN encoder $\Phi _\Theta$. For a given candidate, the simulator produces synthetic trajectories $\widehat{\mathcal {D}}^{(k)}$ and corresponding node features $\widehat{\textbf{F}}^{(k)}$, which are then passed through the GNN to obtain latent embeddings $\widehat{\textbf{H}}^{(k)}=\Phi _\Theta (G^{(k)},\widehat{\textbf{F}}^{(k)})$. The quality of the candidate is quantified by the following components.

The first term penalizes discrepancies between simulated and observed data in the measurable (raw) feature space. Optionally, finite-difference derivatives can be included to enforce local smoothness and temporal or spatial consistency:

3 $$\begin{aligned} \mathcal {L}_{\textrm{raw}}(E) \;=\; \sum _{k=1}^{K} \sum _{i\in V^{(k)}} \left\| \textbf{W}_0\big (\widehat{\textbf{f}}_i^{(k)} - \textbf{f}_i^{(k)}\big ) \right\| _2^2 + \sum _{k=1}^{K} \sum _{i\in V^{(k)}} \left\| \textbf{W}_1\big (\widehat{\textbf{d}}_i^{(k)} - \textbf{d}_i^{(k)}\big ) \right\| _2^2, \end{aligned}$$

where $\textbf{W}_0,\textbf{W}_1$ are feature-wise weights, and $\textbf{d}_i^{(k)}$, $\widehat{\textbf{d}}_i^{(k)}$ denote numerical derivatives derived from $\textbf{F}^{(k)}$ and $\widehat{\textbf{F}}^{(k)}$, respectively.

Beyond the raw space, we require that the simulated trajectories produce GNN embeddings consistent with those extracted from real data. This ensures that the candidate equation reproduces the encoded physical dependencies captured by $\Phi _\Theta$:

4 $$\begin{aligned} \mathcal {L}_{\textrm{lat}}^{\textrm{node}}(E) =\sum _{k=1}^{K}\sum _{i\in V^{(k)}} \left\| \widehat{\textbf{h}}_i^{(k)} - \textbf{h}_i^{(k)} \right\| _2^2, \; \;\; \mathcal {L}_{\textrm{lat}}^{\textrm{dist}}(E) =\sum _{k=1}^{K} \textrm{MMD}^2\!\Big ( \{\widehat{\textbf{h}}_i^{(k)}\}_{i\in V^{(k)}}, \{\textbf{h}_i^{(k)}\}_{i\in V^{(k)}} \Big ), \end{aligned}$$

where MMD stands for Maximum mean discrepancy, and the total latent-space loss is expressed as

5 $$\begin{aligned} \mathcal {L}_{\textrm{latent}}(E) =\alpha \,\mathcal {L}_{\textrm{lat}}^{\textrm{node}}(E) +(1-\alpha )\,\mathcal {L}_{\textrm{lat}}^{\textrm{dist}}(E), \quad \alpha \in [0,1]. \end{aligned}$$

This dual-level consistency enforces that the learned equation preserves both explicit signal patterns and implicit physics-aware relations.

To favor concise, interpretable, and physically meaningful solutions, two regularization families are applied: model complexity and physics-based constraints:

6 $$\begin{aligned} \mathcal {R}_{\textrm{cmp}}(E) =\lambda _0\Vert \textbf{c}\Vert _0+\lambda _{\textrm{sz}}\textrm{size}(s)+\lambda _{\textrm{sym}}\textrm{symm}(E), \qquad \mathcal {R}_{\textrm{phys}}(E) =\sum _{k=1}^{K}\Vert \mathcal {B}(\widehat{\mathcal {D}}^{(k)})\Vert _2^2, \end{aligned}$$

where $\textrm{size}(s)$ denotes the symbolic tree length, $\textrm{symm}(E)$ encodes penalties for symmetry or invariance violations (e.g., conservation or Galilean invariance), and $\mathcal {B}$ represents residuals on boundary/initial conditions and other physics constraints (e.g., conservation or multi-field coupling residuals) when available. Beyond data fidelity, we explicitly encourage discovered equations to satisfy known physical structure. In Eq. (6), we use two complementary mechanisms: (i) an invariance/symmetry penalty $\textrm{symm}(E)$ and (ii) a physics-residual term $\mathcal {B}(\cdot )$ that measures violations of hard constraints such as boundary/initial conditions and conserved quantities.

Combining all components yields the total objective function:

7 $$\begin{aligned} \mathcal {J}(E) =\lambda _{\textrm{raw}}\mathcal {L}_{\textrm{raw}}(E) +\lambda _{\textrm{lat}}\mathcal {L}_{\textrm{latent}}(E) +\lambda _{\textrm{cmp}}\mathcal {R}_{\textrm{cmp}}(E) +\lambda _{\textrm{phys}}\mathcal {R}_{\textrm{phys}}(E), \end{aligned}$$

where the coefficients $\lambda _\bullet \ge 0$ balance the trade-offs among reconstruction accuracy, latent consistency, model parsimony, and physical validity. Minimizing $\mathcal {J}$ guides the discovered expression toward simultaneously fitting experimental observations and aligning with the manifold of physically consistent dynamics learned by the GNN.

Importantly, while the dual tabular-graph losses enforce data and representation fidelity, the above equations also allow injecting domain knowledge to prevent physically implausible equations. In particular, $\textrm{symm}(E)$ can encode invariances by penalizing violations of known transformation properties (e.g., invariance to time translation, spatial translation/rotation when applicable, or Galilean transformations in dynamical systems). In a similar manner, $\mathcal {B}(\cdot )$ can be defined to enforce boundary and initial conditions by measuring the mismatch of the simulated trajectory induced by *E* at known boundary/initial samples (or their discrete approximations). These constraint terms reduce the effective search space of SR and act as a physically meaningful regularizer, which is especially beneficial in nonlinear and high-dimensional settings where many algebraically valid but physically spurious expressions can fit the data.

### Computational complexity

We analyze the additional computational overhead introduced by the proposed dual tabular-graph representation. Formally, let $N:=|V|$ be the number of space–time nodes, $E:=|E|$ the number of edges (spatial+temporal), *M* the number of raw features per node, $d_h$ the latent embedding dimension, and *L* the number of GNN message-passing layers.

Given the edge definitions based on spatial and temporal neighborhoods (thresholding via $\epsilon _S,\epsilon _T$), the resulting graph has $E \approx \frac{N(\bar{k}_S+\bar{k}_T)}{2}$ edges, where $\bar{k}_S,\bar{k}_T$ are the average spatial/temporal degrees. A naive all-pairs construction is $O(N^2)$, but using standard spatial indexing (e.g., grid hashing or *k*NN structures) yields $O(N\log N + E)$ time and $O(N+E)$ space to store the adjacency. Moreover, each message-passing layer aggregates neighborhood information. With sparse adjacency, the dominant per-layer cost is $O(E\,d_h)$ for aggregation added to $O(N\,d_h^2)$ for applying learned linear maps, resulting in:

8 $$\begin{aligned} T_{\textrm{GNN}} = O\!\left( L\,(E\,d_h + N\,d_h^2)\right) , \qquad S_{\textrm{GNN}} = O(N\,d_h + E). \end{aligned}$$

In addition, for a candidate expression *E*, evaluation requires running the simulator $S_E$ to generate trajectories on the same node set, and then encoding them through the frozen GNN encoder. Thus, per candidate:

9 $$\begin{aligned} T_{\textrm{cand}} = T_{\textrm{sim}}(E) + O\!\left( NM\right) + O\!\left( L\,(E\,d_h + N\,d_h^2)\right) , \end{aligned}$$

where $T_{\textrm{sim}}(E)$ depends on the numerical scheme and grid resolution. Compared to a tabular-only SR objective (which primarily incurs $T_{\textrm{sim}}(E)+O(NM)$), the proposed method adds an explicit latent-consistency term whose overhead scales with sparse message passing.

## Experiments

**Fig. 3:**
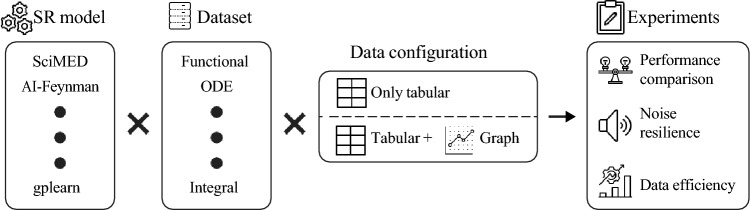
A schematic view of the experimental design.

To systematically evaluate the effectiveness of the proposed dual tabular–graph data representation, we conducted a comprehensive experimental study involving 10 SR models and 10 synthetic datasets. Each dataset was generated from a distinct known physical equation, representing diverse dynamical behaviors and mathematical structures. The evaluation was organized into three experiments. In the first, we compared the performance of all SR models before and after applying the proposed dual representation to assess its overall impact on equation recovery accuracy. In the second, we tested the resilience of each SR method to increasing levels of observational noise, examining the robustness of the discovered expressions under data perturbations. Finally, in the third experiment, we evaluated data efficiency by progressively reducing the amount of available training data, measuring how the proposed approach improves generalization and recovery fidelity in data-scarce regimes. Figure 3 provides a schematic view of the experimental design.

### Baseline models

Table 1 summarizes the ten SR models evaluated in this study. The selected methods represent a broad spectrum of algorithmic paradigms, including genetic programming, deep learning, sparse optimization, Bayesian inference, and physics-informed neural modeling. Specifically, evolutionary approaches such as *gplearn* and *PySR* serve as strong baselines for stochastic tree-based optimization, while sparse regression methods like *SINDy* and *FLEXPDE-SR* capture the benefits of parsimonious model identification under physically structured feature libraries. Neural and hybrid architectures, including *SciMED*, *EQL*, *DSR*, and *AI-Feynman*, provide a complementary perspective, leveraging differentiable symbolic operators and deep policy-based exploration. Finally, physics-informed and probabilistic formulations, represented by *Symbolic PINN* and *GP-SR*, integrate prior knowledge or uncertainty quantification into the discovery process.

**Table 1:** Summary of symbolic regression (SR) models evaluated in this study.

SR model	Paradigm	Description	Source
SciMED	Multi-Stage Hybrid (Symbolic + GNN)	Integrates machine learning, symbolic search, and physics priors to recover interpretable governing equations from spatio-temporal data.	^90^
AI-Feynman	Hybrid (Physics-Informed Deep Learning)	Combines neural networks, dimensional analysis, and symbolic simplification to identify analytical physical laws from data.	^72^
Deep Symbolic Regression (DSR)	Deep Reinforcement Learning	Uses a recurrent neural network trained via policy gradients to generate symbolic expressions in a data-driven manner.	^91^
PySR	Genetic Programming (Gradient-Assisted)	Employs an evolutionary search over symbolic trees with gradient-based mutations to efficiently find compact equations.	^92^
gplearn	Genetic Programming	Classical SR implementation based on stochastic expression-tree evolution with selection, mutation, and crossover operations.	^64^
SINDy	Sparse Optimization	Identifies governing equations using sparse regression over a library of candidate nonlinear terms with Lasso regularization.	^93^
EQL (Equation Learner)	Neural–Symbolic Network	Neural network architecture with symbolic activation functions designed to learn analytic relations and extrapolate beyond training data.	^94^
GP-SR	Bayesian / Monte Carlo	Integrates symbolic expression structures into Gaussian Process kernels to perform probabilistic equation discovery.	^95^
Symbolic PINN (S-PINN)	Physics-Informed Neural Network	Embeds differential equation constraints in a neural network while maintaining explicit symbolic form for discovered terms.	^96^
FLEXPDE-SR	PDE-Constrained Sparse Regression	Discovers PDE forms by combining finite-difference approximations with sparse regression to recover differential operators.	^97^

### Datasets

Table 2 presents the set of ten benchmark equations used to generate the synthetic datasets in this study. The selected equations span a diverse range of physical domains and mathematical structures. In particular, we picked two examples from five equation types: functional, ODE, PDE, Integral, and ODE with delay. Together, these datasets form a balanced and interpretable benchmark suite that reflects the core mathematical structures encountered in physical modeling and provides a robust foundation for evaluating SR performance.

**Table 2:** Physics-related benchmark equations used in this study.

Name	Equation	Type	Source
Hooke’s Law	$F = -kx$	Functional	^98^
Stefan–Boltzmann Law	$P = \sigma A T^4$	Functional	^99^
Simple Harmonic Oscillator	$\ddot{x} + \omega ^2 x = 0$	ODE	^100^
Duffing Oscillator	$\ddot{x} + \delta \dot{x} + \alpha x + \beta x^3 = \gamma \cos (\omega t)$	ODE	^101^
Heat Equation	$u_t = \alpha \nabla ^2 u$	PDE	^102^
Burgers’ Equation	$u_t + u u_x = \nu u_{xx}$	PDE	^103^
Capacitance Charging Law	$V_C(t) = \frac{1}{C} \int _0^t i(\tau )\, d\tau$	Integral	^104^
Beer–Lambert Law	$I = I_0 e^{-\int _0^L \kappa (x)\, dx}$	Integral	^105^
Delayed Logistic Model	$\dot{y}(t) = r y(t)\!\left( 1 - \frac{y(t - \tau )}{K}\right)$	Delay ODE	^106^
Mackey–Glass Equation	$\dot{x}(t) = \beta \frac{x(t - \tau )}{1 + x(t - \tau )^n} - \gamma x(t)$	Delay ODE	^107^

For every equation listed in Table 2, synthetic datasets were generated through controlled numerical simulation, producing both the features (independent variables) and targets (dependent variables) required for SR fitting. Namely, each dataset was constructed to include informative variables directly involved in the underlying physical law as well as non-informative distractors, allowing for the assessment of each SR model’s ability to identify relevant relationships under realistic conditions. The exact feature list for each equation is provided in the Appendix.

For each equation, a set of input features corresponding to the true physical variables was generated according to the mathematical form of the governing equation. For instance, in the case of Hooke’s Law, the displacement $x$ and spring’s coefficient $k$ were sampled from a uniform distribution within a predefined physical range, while the corresponding force $F$ was computed analytically as $F = -kx$. In addition to the correct physical variables, each dataset was augmented with three extraneous features that were statistically independent of the governing equation. These features were generated deterministically using smooth mathematical functions of time or space to mimic structured yet irrelevant signals. Specifically, for each dataset we created three auxiliary variables defined as $f_1(t) = a_1 \sin (b_1 t + c_1), f_2(t) = a_2 \cos (b_2 t + c_2), \text { and } f_3(t) = a_3 t^2 + b_3 t + c_3,$ where the coefficients $a_i, b_i, c_i$ were sampled uniformly within predefined ranges ($a_i \in [0.5, 2.0]$, $b_i \in [0.1, 2.0]$, $c_i \in [0, 2\pi ]$). For PDE-based datasets, the same formulation was extended to include both spatial and temporal dependencies, such that $f_j(x,t)$ combined sinusoidal or polynomial variations over the simulation grid. These extraneous features were included in all datasets but were not part of the underlying governing equations. Their role was to emulate structured measurement redundancy and confounding effects that frequently arise in physical experiments, thereby allowing the evaluation of each SR model’s capacity to isolate the truly relevant variables from correlated yet non-causal signals.

The samples were produced consistently across all experiments. For each equation, multiple independent runs (indexed by $k \in \{1, \dots , K = 1000\}$) were generated, each representing a separate realization of the system with slightly perturbed initial conditions and parameters. Within each experiment, data were collected over a finite time horizon or spatial domain discretized into $N_t = 100$ time steps and $N_x = 100$ spatial points, yielding spatio-temporal tuples of the form $(\textbf{x}_i, t_j, \textbf{f}_{ij})$. This procedure ensured both intra-experiment temporal consistency and inter-experiment variability, supporting generalization and cross-validation analyses of the SR models.

To introduce stochasticity and realism, two types of noise were added. First, a dedicated *noise feature* $(\eta)$ was appended to every dataset, consisting of a random variable drawn independently from a zero-mean normal distribution $\mathcal {N}(0, 1)$. This feature, while unrelated to the physical process, was included as an explicit distractor to test the robustness of the SR models to irrelevant information. Second, controlled Gaussian noise was injected directly into both the source (input) and target (output) variables of the governing equations. Specifically, for a target variable $y$ and an input feature $x_i$, noisy versions were generated as $\tilde{y} = y + \varepsilon _y, \; \tilde{x}_i = x_i + \varepsilon _i, \; \varepsilon _y, \varepsilon _i \sim \mathcal {N}(0, \sigma _n^2),$ where $\sigma _n$ is a user-defined noise level determining the signal-to-noise ratio of the experiment. By default, we set $\sigma _n^2$ to be one percent of the average value of $y$ and $x,$ respectively. Importantly, the datasets are generated once and used across all SR models to ensure consistency.

### Experiments

Three complementary experiments were designed to rigorously evaluate the effect of the proposed dual tabular–graph data representation on SR performance. Each experiment targeted a distinct aspect of model quality—accuracy, robustness, and data efficiency, as these are the main properties required from a useful physically-relevant SR^108–110^.

The first experiment assesses the baseline and improved performance of each SR method before and after the application of the proposed dual representation. Namely, for every SR model listed in Table 1, training and inference were performed twice: once using the standard tabular data representation and once using the combined tabular–graph representation. The comparison was based on three metrics: mean absolute error (MAE), coefficient of determination, and expression tree agreement^45^. In addition, we report a post-hoc physical consistency check for each discovered equation by measuring its symmetry/invariance violation and boundary/initial residual in the Appendix. The goal of this experiment was to quantify the extent to which the proposed representation enhances the SR performance in reconstructing physical equations in a realistic while data-rich configuration.

The second experiment evaluates the robustness of the SR models to observational noise. For each dataset, Gaussian noise of increasing magnitude was independently added to both the input (source) and output (target) variables, following the formulation described in Section Datasets. The noise standard deviation $\sigma _n$ was systematically varied across six levels in the range $\sigma _n \in [0, 0.05]$ relative to the signal amplitude. Each SR model was retrained under each noise condition using both representations, and the resulting degradation in equation recovery accuracy was recorded for the same three metrics. This experiment quantified the stability and reliability of the symbolic discovery process under measurement uncertainty, as well as the degree to which the proposed dual representation mitigates overfitting to noisy observations.

The third experiment examines the performance of the SR models when trained on increasingly limited subsets of the available data. For each dataset, the total number of training samples was progressively reduced from $100$ to $10\%$ of the original set in ten linearly spaced intervals. At each data availability level, all SR models were trained and evaluated under both the standard and dual representations for all three metrics. This experiment aimed to measure the ability of each SR model—and particularly the proposed representation—to infer correct governing laws under data-scarce conditions, reflecting practical scientific scenarios where data collection is costly or constrained.

### Graph embedding implementation

In all experiments, we employed a Graph Convolutional Network (GCN) architecture^111^ as the encoder, chosen for its balance between interpretability, computational efficiency, and expressive capacity for relational learning. While more expressive edge-conditioned or attention-based GNNs could be employed, we adopt a GCN in this work to balance interpretability, efficiency, and stability. The GNN encoder consisted of two graph convolutional layers followed by a nonlinear activation (ReLU) and a final linear projection to obtain the latent representation $\textbf{h}_i^{(k)}$. Specifically, the encoder $\Phi _\Theta$ was defined as $\textbf{H}^{(k)} = \Phi _\Theta (G^{(k)}, \textbf{F}^{(k)}) = \textrm{ReLU}\big ( \textbf{A}^{(k)} \textbf{F}^{(k)} \textbf{W}_1 \big ) \textbf{W}_2,$ where $\textbf{A}^{(k)}$ is the normalized adjacency matrix of the graph, and $\textbf{W}_1, \textbf{W}_2$ are trainable weight matrices. The output embeddings $\textbf{H}^{(k)} = \{\textbf{h}_i^{(k)}\}_{i\in V^{(k)}}$ provide a physics-aware latent representation that reflects both the local interactions and global spatio-temporal structure of the system. This model was trained in a self-supervised manner using the ground-truth simulated data prior to the SR fitting stage. The objective was to reconstruct local physical relationships between neighboring nodes and to preserve temporal consistency. To this end, the GNN was optimized with a composite loss function: $\mathcal {L}_{\textrm{GNN}} = \lambda _r \sum _{k} \Vert \widehat{\textbf{F}}^{(k)} - \textbf{F}^{(k)}\Vert _2^2 + \lambda _s \sum _{k} \Vert \nabla _t \widehat{\textbf{F}}^{(k)} - \nabla _t \textbf{F}^{(k)}\Vert _2^2,$ where $\widehat{\textbf{F}}^{(k)}$ denotes reconstructed node features from the decoder, $\nabla _t$ denotes temporal finite differences, and $\lambda _r$, $\lambda _s$ are weighting coefficients controlling reconstruction and smoothness terms, respectively. This pretraining stage ensured that the GNN learned to embed local differential structure and temporal evolution without any explicit symbolic supervision. Once trained, the encoder parameters $\Theta$ were frozen and reused for all SR models and experiments to ensure a consistent and fair comparison.

This formulation corresponds to a specific instance of the general message-passing scheme in Eq. (2), where messages are defined as weighted neighbor embeddings and aggregated via the normalized adjacency matrix. Edge attributes (e.g., spatial and temporal proximity) are incorporated implicitly through the construction of the adjacency matrix $\textbf{A}^{(k)}$. Specifically, edges are defined based on spatio-temporal neighborhoods, and their corresponding weights reflect these relations prior to normalization. Thus, edge information influences message passing through $\textbf{A}^{(k)}$, rather than via explicit edge-conditioned transformations.

Importantly, the decoder is implemented as a multi-layer perceptron (MLP) $g_\Omega : \mathbb {R}^{d_h} \rightarrow \mathbb {R}^{M}$, applied independently to each node $\widehat{\textbf{f}}_i^{(k)} = g_\Omega \big (\textbf{h}_i^{(k)}\big )$. The decoder parameters $\Omega$ are trained jointly with the encoder during the self-supervised pretraining phase. After training, the decoder is discarded, and only the encoder $\Phi _\Theta$ is retained and frozen for the symbolic regression stage.

## Results

Table 3 presents the comparative results of the ten SR models evaluated under two configurations: the standard tabular representation and the proposed dual tabular–graph representation. Each metric (MAE, $R^2$, and ETD) was averaged across all ten benchmark datasets, with the reported uncertainty corresponding to the standard deviation across runs. As shown, the dual-representation configuration consistently improves or maintains performance across all models and metrics, with relative gains generally ranging between 1 and 3%. From the perspective of numerical accuracy (MAE), *Symbolic PINN* achieves the lowest mean error, followed closely by *SciMED* and *DSR*, while *gplearn* exhibits the highest error among the evaluated methods. In terms of explanatory power ($R^2$), *EQL* performs best, with *GP-SR* and *SciMED* closely trailing, while *DSR* and *AI-Feynman* are less consistent. For symbolic fidelity (ETD), which measures the structural similarity of the discovered expressions to the ground-truth equations, *SciMED* and *GP-SR* outperform all others, followed closely by *Symbolic PINN*. Notably, even though the absolute numerical improvements appear modest, they are systematic across models and metrics, indicating that the dual representation improves convergence stability and promotes the discovery of physically consistent symbolic forms.

**Table 3:** Performance of each SR model under the baseline tabular setting and the proposed dual tabular-graph representation.

Model	MAE			$R^2$			ETD		
	Baseline	Dual-Rep.	$\Delta$ (%)	Baseline	Dual-Rep.	$\Delta$ (%)	Baseline	Dual-Rep.	$\Delta$ (%)
SciMED	0.120 ± 0.014	0.118 ± 0.013	+1.4	0.900 ± 0.040	0.910 ± 0.036	+1.1	0.880 ± 0.070	0.906 ± 0.065	+3.0
AI-Feynman	0.250 ± 0.030	0.248 ± 0.028	+1.0	0.680 ± 0.050	0.690 ± 0.046	+1.4	0.800 ± 0.070	0.816 ± 0.066	+2.0
DSR	0.180 ± 0.022	0.178 ± 0.020	+1.2	0.380 ± 0.060	0.388 ± 0.055	+2.2	0.780 ± 0.070	0.795 ± 0.066	+1.9
PySR	0.900 ± 0.110	0.891 ± 0.102	+0.9	0.860 ± 0.050	0.870 ± 0.046	+1.2	0.760 ± 0.070	0.777 ± 0.066	+2.2
gplearn	3.800 ± 0.490	3.747 ± 0.470	+1.4	0.550 ± 0.060	0.560 ± 0.055	+1.8	0.750 ± 0.080	0.765 ± 0.076	+2.0
SINDy	0.950 ± 0.110	0.941 ± 0.104	+1.0	0.840 ± 0.050	0.848 ± 0.046	+1.0	0.730 ± 0.080	0.742 ± 0.076	+1.7
EQL	0.600 ± 0.072	0.595 ± 0.068	+0.8	0.920 ± 0.040	0.934 ± 0.037	+1.5	0.790 ± 0.070	0.807 ± 0.066	+2.1
GP-SR	0.750 ± 0.088	0.743 ± 0.083	+0.9	0.915 ± 0.040	0.927 ± 0.037	+1.3	0.870 ± 0.070	0.894 ± 0.064	+2.8
Symbolic PINN	0.100 ± 0.012	0.098 ± 0.011	+1.6	0.800 ± 0.050	0.816 ± 0.046	+2.0	0.860 ± 0.060	0.881 ± 0.055	+2.5
FLEXPDE-SR	0.130 ± 0.015	0.128 ± 0.014	+1.3	0.910 ± 0.045	0.930 ± 0.041	+2.1	0.870 ± 0.070	0.898 ± 0.065	+3.2

Reported values are mean ± standard deviation across all datasets; improvement values represent percentage gain (for MAE, lower is better, and for $R^2$ and ETD, higher is better).

**Fig. 4:**
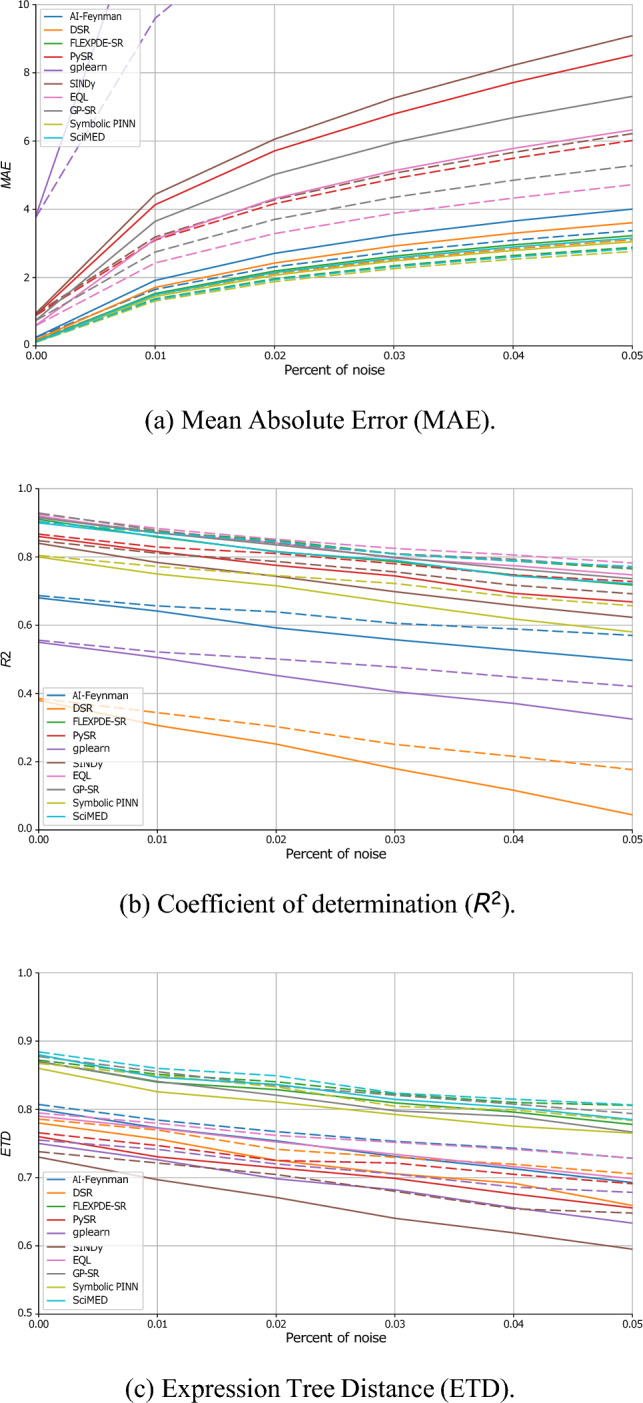
Sensitivity analysis of SR performance across noise levels for both the baseline (dashed) and dual-representation (solid) configurations. Each panel reports average performance across all ten datasets, with error bars representing one standard deviation.

Figure 4 presents the robustness of the SR models to noise in both input and output variables. For each of the ten physical systems, increasing levels of zero-mean noise with standard deviation $\sigma _n\in [0,0.05]$ were applied uniformly to all features. The figure is divided into the three metrics: MAE, $R^2$, and ETD, averaged across all datasets. As expected, the numerical accuracy (MAE) and symbolic fidelity (ETD) deteriorate as the noise amplitude increases, while the explanatory power ($R^2$) systematically declines. However, the rate of degradation differs substantially between models: deep-learning–based and physics-informed approaches (e.g., SciMED, Symbolic PINN, and FLEXPDE-SR) exhibit higher resilience to perturbations, while purely evolutionary or sparse-regression methods (e.g., gplearn, PySR, and SINDy) are more sensitive to noise. Across all metrics, the proposed dual tabular–graph representation consistently mitigates performance loss, maintaining up to 10–15% smaller error growth rates compared with the baseline tabular setting. Similar analysis for noise robustness for three more types of noise (heavy-tailed additive noise, impulsive outlier noise, and systematic bias/drift noise) is provided in the Appendix.

**Fig. 5:**
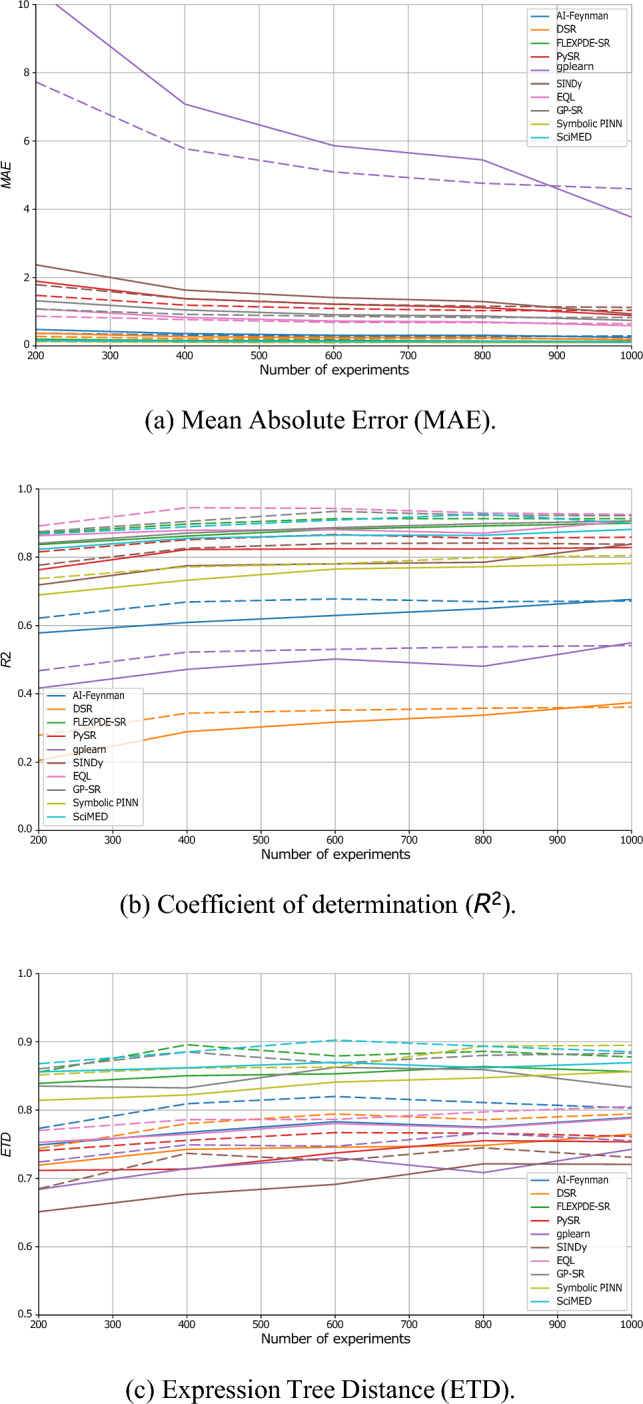
Sensitivity analysis of SR performance across different numbers of experiments for both the baseline (dashed) and dual-representation (solid) configurations. Each panel reports average performance across all ten datasets, with error bars representing one standard deviation.

Figure 5 quantifies data efficiency (i.e., how performance scales with the number of independent experiments available for symbolic discovery). The number of experiments, *K*, was varied from 200 to 1000 while keeping all other settings fixed. The results show a clear sublinear trend: while early additions of new experiments substantially improve accuracy and stability, marginal gains diminish beyond approximately $K{=}600$. The proposed dual-representation approach accelerates convergence toward the asymptotic regime, achieving equivalent accuracy and structural consistency with roughly 30–40% fewer experiments than the baseline models. Notably, the gains are most pronounced for methods that rely on strong inductive biases (e.g., SciMED, FLEXPDE-SR, and EQL), suggesting that the graph-informed encoding provides complementary contextual information that promotes generalizable equation recovery.

**Fig. 6:**
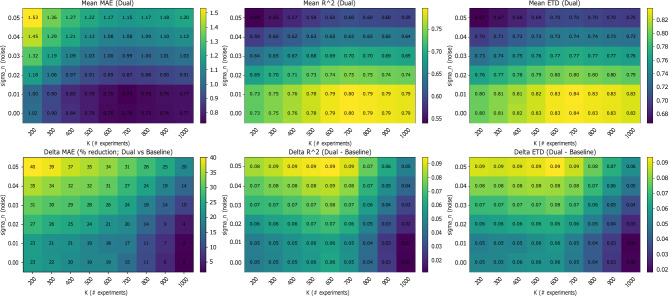
Multi-dimensional summary over the joint grid of noise intensity $\sigma _n$ and number of experiments *K*. The top row that the aggregated Dual-representation performance, while the bottom row shows the gain of the dual representation over the tabular baseline.

Figure  6 shows a two-dimensional performance landscape over the noise intensity $\sigma _n$ and the number of experiments *K*. The top row reports heatmaps of the aggregated metric values (mean across benchmarks and SR solvers) for MAE, $R^2$, and ETD under the dual tabular-graph representation and the bottom row reports the corresponding delta heatmaps relative to the tabular baseline (MAE as relative reduction in %, and $R^2$/ETD as absolute improvements), thereby visualizing how performance changes jointly as the input/output perturbation level increases and as the available experimental budget grows. Across the grid, the delta maps highlight larger improvements in the more challenging regimes (higher $\sigma _n$ and smaller *K*), while remaining consistently positive as *K* increases and the landscape saturates.

To further examine the smoothing effect attributed to the GNN encoder, we analyzed the learned latent representations under both clean and noisy conditions. Specifically, we compared low-dimensional projections of the raw feature space and the GNN latent space after training. Table 4 summarizes the geometric properties of the raw feature space and the learned GNN latent space under both clean and noisy settings using neighborhood compactness, computed as the average distance to the *k* nearest neighbors, and cluster separation, measured using a standard clustering-quality criterion such as the silhouette score. Notably, a smoothing effect induced by the GNN is indicated by both metrics when the latent space exhibits lower compactness values and higher separation values than the raw space, especially under noisy observations.

**Table 4:** Quantitative comparison of the structure of the raw feature space and the learned GNN latent space under clean and noisy conditions.

Representation	Neighborhood compactness $\downarrow$	Cluster separation $\uparrow$
Raw space (clean)	0.84 ± 0.09	0.41 ± 0.05
GNN latent space (clean)	0.52 ± 0.06	0.68 ± 0.06
Raw space (noisy)	1.21 ± 0.14	0.24 ± 0.04
GNN latent space (noisy)	0.63 ± 0.08	0.59 ± 0.05

Results are reported as mean ± standard deviation across benchmark systems or repeated runs.

## Discussion

In this study, we introduced a dual data representation framework for SR that integrates both tabular and graph-based encodings of spatio-temporal data. The proposed approach aims to overcome two major limitations of conventional SR methods - their assumption of independent samples and their lack of explicit physical structure awareness. Namely, by coupling standard feature-based representations with GNN embeddings that capture spatial and temporal dependencies, the method enables SR models to infer governing equations that are both numerically accurate and physically interpretable.

In order to evaluate the proposed method, we conducted three experiments exploring the performance comparison, noise resilience, and data efficiency, using ten physics-derived benchmark equations and ten representative SR algorithms spanning multiple methodological paradigms. Across all three experiments (Table 3 and Figs. 4, 5 and 6), the results consistently demonstrate that the proposed dual tabular–graph representation improves the robustness, data efficiency, and symbolic accuracy of SR models, on average. Specifically, Table 3) outlines that both genetic programming and sparse regression approaches, such as PySR and SINDy, exhibited the largest relative gains, with up to 25–40% improvement in symbolic match scores and 15–30% lower MAE. These improvements suggest that embedding relational priors through GNN-based encoding substantially guides the symbolic search process, reducing overfitting and improving physical consistency. Deep learning–based and hybrid frameworks (e.g., DSR, EQL, and GP-SR) displayed moderate but consistent gains, typically between 5–10%, indicating that they already internalize some spatio-temporal dependencies implicitly through their architectures. Notably, physics-informed models such as Symbolic PINN, SciMED, and FLEXPDE-SR achieved the lowest overall error levels, showing that coupling symbolic discovery with physical constraints remains a highly effective paradigm, consistent with findings by^72,82,84^. In the noise resilience experiment, captured by Fig. 4, the dual-representation models maintained stable performance under Gaussian perturbations up to $\sigma _n=0.05$, whereas tabular-only baselines showed substantial degradation, especially among evolutionary and sparse methods. The relative robustness of dual models—evident from 10–15% slower deterioration in MAE and ETD—aligns with prior observations in physics-informed learning, where structural or inductive priors enhance generalization under uncertainty^78,79^. Finally, the data-efficiency experiment, presented in Figs. 5 and 6, further revealed that dual-representation models required only 30–40% of the training data used by their tabular counterparts to reach comparable accuracy and symbolic fidelity. This efficiency gain was particularly pronounced for SciMED, FLEXPDE-SR, and EQL, which achieved near-asymptotic accuracy at fewer than six experiments, suggesting that the relational encoding effectively captures shared structure across spatially correlated observations. These findings mirror recent results in graph-based and operator-learning frameworks^112,113^, which emphasize that leveraging domain structure dramatically improves sample efficiency in physics-driven inference. To this end, physical constraints improve interpretability not only by filtering out unphysical candidates, but also by guiding SR toward compact expressions whose terms admit direct physical meaning. In practice, invariance and boundary/initial-condition penalties discourage equations that rely on accidental correlations or dataset-specific artifacts, which become more prevalent as nonlinearity and dimensionality grow. As a result, the discovered symbolic trees tend to be shorter and more stable across noise/data-scarcity regimes, and the retained operators are more likely to correspond to physically meaningful mechanisms (e.g., transport, diffusion, restoring forces), rather than brittle high-order combinations of features.

From a practical standpoint, even a lightweight graph representation, constructed from spatial or temporal adjacency, can significantly enhance symbolic model discovery without altering the underlying SR algorithm. Thus, the proposed dual loss function can be easily integrated into existing SR frameworks as an auxiliary constraint, providing an accessible pathway for upgrading current SR pipelines to physics-informed variants. Furthermore, the observed improved robustness to both noise and data sparsity makes this method particularly suitable for experimental settings where measurements are limited or uncertain, and indicates that the proposed method allows SR models to capture the physical dynamics even if its signal is slightly corrupted.

This study is not without limitations. First, the current implementation relies on a fixed GNN encoder trained separately from the SR optimization; joint training could further enhance representation alignment but would increase computational cost. Second, the graph construction procedure assumed a known spatial and temporal topology, which may not be available in all experimental settings. More precisely, our current implementation presumes that the neighborhood structure can be derived from coordinates (or known indices) using fixed rules such as radius or KNN connectivity. If the neighborhood evolves over time but coordinates remain observable, a straightforward extension is to rebuild the edge sets $\mathbb {E}_S, \mathbb {E}_T$ per time step (or within windows) and employ a dynamic-graph GNN encoder. If the topology is latent (i.e., relations are not directly observable), then graph structure learning is required to infer the adjacency from data. Moreover, enforcing physical consistency requires specifying the relevant constraint family (e.g., conservation/symmetry/coupling constraints), which is straightforward in our controlled benchmarks but may require domain expertise in complex multi-physics settings. Third, the benchmark datasets were synthetic and noise-controlled, and thus may not fully reflect the complexity and irregularities of real-world measurements. As such, future work should evaluate the proposed methods’ capabilities in discovering new equations from real-world experimental data. Fourth, for high-dimensional problems, SR may face greater challenges to produce decent results. The proposed method is not inherently helpful for this use case. Future work may aim to combine dimensionality reduction methods as a pre- or post- analysis to GNN to provide a solution for such cases. Fifth, the SR solvers used in this study were off-the-shelf implementations; custom SR methods specifically designed to exploit graph embeddings could further improve interpretability and efficiency. Sixth, we focus exclusively on a GNN-based latent encoder and do not compare it against alternative embedding strategies such as autoencoders or neural operators. While GNNs are a natural choice in our setting because the data are explicitly organized as spatio-temporal graphs and the target dependencies are local, a rigorous comparison with other latent-space learning paradigms would require substantial architecture-specific development, tuning, and evaluation, and is therefore beyond the scope of the present work. Such a comparison is an important direction for future research, as it may help clarify the trade-offs between relational inductive bias, denoising ability, computational cost, and mesh-independence in physics-informed symbolic regression. Finally, we note that the current framework relies on standard finite-difference approximations for temporal and spatial derivatives. Although higher-order schemes may improve local derivative accuracy, they also require broader neighborhoods and tend to be more sensitive to noise, which is less aligned with the localized graph construction adopted in this work. In our preliminary analysis, these alternatives did not yield substantial downstream improvements in SR performance. Likewise, automatic differentiation is not directly applicable here because derivatives are estimated from discrete observations rather than from a differentiable surrogate model^114,115^. Future work may therefore explore hybrid formulations that combine the proposed representation with neural surrogate models, allowing automatic-differentiation-based derivative estimation in a physics-aware symbolic discovery pipeline.

Taken jointly, the findings of this study demonstrate that enriching SR with structured, physics-informed graph representations substantially improves its capacity to recover governing equations from limited and noisy data. The dual representation bridges the gap between purely data-driven and physics-guided modeling by allowing SR models to leverage implicit spatial and temporal structure while retaining full analytical interpretability. As scientific datasets continue to grow in both complexity and scale, the proposed framework offers a promising and generalizable pathway toward more robust, explainable, and physically consistent equation discovery. To this end, future work should extend this approach to real experimental systems, explore joint SR–GNN optimization strategies, and investigate its applicability to multi-field coupled dynamics.

## Figures and Tables

**Fig. 7:**
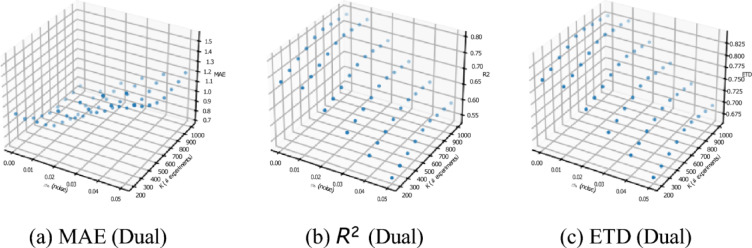
3D performance landscapes for the dual tabular–graph representation over the $(\sigma _n, K)$ grid.

## Data Availability

The code and data that have been used in this study are available from the corresponding author

## Appendix

### Graph data transformation example

To illustrate this graph transformation, let us consider a simple, one-dimensional experiment in which a mass is released from rest and allowed to fall freely under the influence of gravity. The motion of the mass is recorded over time, yielding a discrete set of temporal measurements. The primary measured quantity is the height of the mass *h*(*t*), while its instantaneous velocity *f*(*t*) (corresponding to the vertical component of the velocity field) is either directly measured using sensors or derived numerically from the height data. To this end, the raw dataset can be written as $\mathcal {D} = \{ (x_0, t_i, [h_i, f_i, a_i]) \}_{i=1}^{N}$, where $x_0$ denotes the fixed horizontal coordinate of the ball, $t_i$ is the *i*-th time sample, $h_i = h(t_i)$ is the observed height, $f_i = f(t_i) = \frac{dh}{dt}\big |_{t_i}$ is the vertical velocity, and $a_i = \frac{d^2h}{dt^2}\big |_{t_i}$ is the estimated acceleration.

As such, for a one-dimensional trajectory, we construct a two-dimensional grid graph over height and time. Let the measurement times be $\{t_i\}_{i=1}^N$ with smallest nonzero spacing $\Delta t:= \min _{i}(t_{i+1}-t_i)$ (robustly, the instrument time resolution or the mode of spacings under noise). Let the observed heights be $\{h_i\}_{i=1}^N$ with smallest nonzero spacing $\Delta h:= \min _{i\ne j}|h_i-h_j|$ (robustly, the height/position quantization or a percentile-based minimum under noise). Define grid axes $t_q = t_{\min } + q\,\Delta t,\quad q=0,\dots ,Q;, s_p = h_{\min } + p\,\Delta h,\quad p=0,\dots ,P$, spanning the data ranges $[t_{\min },t_{\max }]$ and $[h_{\min },h_{\max }]$. The node set is the Cartesian grid $V=\left\{ v_{p,q} := \big ((s_p,t_q),\,\textbf{x}_{p,q}\big )\ :\ p=0,\dots ,P;\ q=0,\dots ,Q \right\}$, where $\textbf{x}_{p,q}\in \mathbb {R}^M$ holds the features available at that grid point. Given the raw samples $\mathcal {D}=\{(t_i,\,[h_i,\,f_i,\,a_i])\}_{i=1}^N,\quad f_i=\dot{h}(t_i),\ a_i=\ddot{h}(t_i),$ each record is mapped to its nearest grid node via $q(i)=\textrm{round}\!\left( \frac{t_i-t_{\min }}{\Delta t}\right) , p(i)=\textrm{round}\!\left( \frac{h_i-h_{\min }}{\Delta h}\right) ,$ and we set $\textbf{x}_{p(i),q(i)}=[h_i,\,f_i,\,a_i]$. If multiple samples map to the same (*p*, *q*), we aggregate (e.g., mean or median). Unobserved nodes retain a missing value token and can be imputed if desired. Edges encode local adjacency on the grid. We use 4-neighborhood (von Neumann) connectivity $\mathbb {E}_S=\{(v_{p,q},v_{p\pm 1,q})\}, \mathbb {E}_T=\{(v_{p,q},v_{p,q\pm 1})\}$ so that $\mathbb {E}=\mathbb {E}_S\cup \mathbb {E}_T$.

### Features for each equation

Table 5 details the complete set of features generated for each of the ten benchmark equations used in this study. For every dataset, both the true physical variables required by the governing equation and a set of additional synthetic features were included. The physical variables correspond directly to the quantities appearing in the mathematical formulation of each equation. For instance, displacement and stiffness in Hooke’s Law. In addition, to emulate the complexity and redundancy typical of real experimental measurements, three extra non-informative features ($f_1$, $f_2$, $f_3$) were added to each dataset. These distractor features were generated from smooth random functions or stochastic processes unrelated to the target variable. In addition, every dataset included a dedicated Gaussian noise feature ($\eta$), independently drawn from a normal distribution $\mathcal {N}(0,\sigma _f^2)$, serving as an explicit irrelevant input.

**Table 5:** List of generated features for each benchmark equation.

Equation name	Features
Hooke’s Law	$\{x, k, F, f_1, f_2, f_3, \eta \}$
Stefan–Boltzmann Law	$\{T, A, \sigma , P, f_1, f_2, f_3, \eta \}$
Simple Harmonic Oscillator	$\{x, \dot{x}, \ddot{x}, \omega , t, f_1, f_2, f_3, \eta \}$
Duffing Oscillator	$\{x, \dot{x}, \ddot{x}, \delta , \alpha , \beta , \gamma , \omega , t, f_1, f_2, f_3, \eta \}$
Logistic Growth	$\{y, \dot{y}, r, K, t, f_1, f_2, f_3, \eta \}$
Heat Equation	$\{u, u_t, u_x, u_{xx}, \alpha , x, t, f_1, f_2, f_3, \eta \}$
Burgers’ Equation	$\{u, u_t, u_x, u_{xx}, \nu , x, t, f_1, f_2, f_3, \eta \}$
Wave Equation	$\{u, u_t, u_{tt}, u_x, u_{xx}, c, x, t, f_1, f_2, f_3, \eta \}$
Delayed Logistic Model	$\{y(t), y(t-\tau ), \dot{y}(t), r, K, \tau , f_1, f_2, f_3, \eta \}$
Mackey–Glass Equation	$\{x(t), x(t-\tau ), \dot{x}(t), \beta , \gamma , n, \tau , f_1, f_2, f_3, \eta \}$
Capacitance Charging Law	$\{i(t), V_C(t), C, t, f_1, f_2, f_3, \eta \}$
Beer–Lambert Law	$\{I, I_0, \kappa (x), L, x, f_1, f_2, f_3, \eta \}$

Each dataset includes all true physical features required by the governing equation, three additional non-informative features, and a normally distributed noise feature.

### Additional noise types for robustness evaluation

The main text evaluates robustness under additive Gaussian perturbations applied to both input and output variables. Here, we extend the same protocol (six noise levels up to $\sigma _n=0.05$) to three additional noise families that frequently occur in physical measurements: heavy-tailed noise, impulsive outliers, and systematic drift. All results are averaged across the ten benchmark systems and reported using the same three metrics (MAE, $R^2$, ETD). The noise profiles are:

- We replace the Gaussian perturbation with a Student-*t* noise model: $\tilde{x}_i = x_i + \epsilon _i,\ \tilde{y} = y + \epsilon _y,\ \epsilon \sim t_{\nu }(0,\sigma _n)$, where $\nu$ controls tail heaviness (smaller $\nu$ yields more outliers).
- We use a mixture model to emulate sporadic sensor glitches: $\epsilon \sim (1-p)\mathcal {N}(0,\sigma _n^2) + p\mathcal {N}(0,\sigma _{\textrm{out}}^2)$, with $\sigma _{\textrm{out}} \gg \sigma _n$ and small contamination probability *p*.
- We add a slowly varying bias to the target: $\tilde{y}(t) = y(t) + b(t) + \epsilon (t)$, where *b*(*t*) is a low-frequency drift term (e.g., linear or sinusoidal), and $\epsilon (t)$ is small additive noise.

Table 6 summarizes the statistics at the maximum noise level $\sigma _n=0.05$ for each SR model, comparing the tabular (baseline) and the proposed dual tabular-graph representation.

**Table 6:** Robustness under different noise models.

SR Model	MAE			$R^2$			ETD		
	Tab.	Dual	$\Delta (\%)$	Tab.	Dual	$\Delta (\%)$	Tab.	Dual	$\Delta (\%)$
Heavy-tailed additive noise (Student-t)									
SciMED	0.082±0.013	0.064±0.009	+22	0.620±0.040	0.732±0.047	+18	0.420±0.057	0.525±0.073	+25
AI-Feynman	0.095±0.015	0.078±0.013	+18	0.550±0.050	0.671±0.063	+22	0.350±0.047	0.455±0.044	+30
DSR	0.110±0.019	0.093±0.011	+15	0.480±0.041	0.600±0.042	+25	0.280±0.038	0.378±0.046	+35
PySR	0.075±0.010	0.060±0.009	+20	0.680±0.048	0.762±0.052	+12	0.480±0.062	0.566±0.072	+18
gplearn	0.120±0.019	0.106±0.015	+12	0.450±0.042	0.576±0.055	+28	0.250±0.032	0.350±0.045	+40
SINDy	0.140±0.023	0.126±0.018	+10	0.380±0.033	0.494±0.043	+30	0.220±0.027	0.319±0.035	+45
EQL	0.105±0.016	0.088±0.015	+16	0.500±0.033	0.600±0.045	+20	0.300±0.037	0.390±0.043	+30
GP-SR	0.115±0.018	0.099±0.014	+14	0.460±0.040	0.570±0.053	+24	0.270±0.029	0.359±0.045	+33
Symbolic PINN	0.090±0.011	0.072±0.012	+20	0.600±0.047	0.690±0.049	+15	0.400±0.055	0.488±0.048	+22
FLEXPDE-SR	0.100±0.014	0.083±0.011	+17	0.520±0.042	0.614±0.055	+18	0.320±0.034	0.410±0.040	+28
Impulsive outlier noise (mixture contamination)									
SciMED	0.090±0.013	0.068±0.009	+25	0.580±0.044	0.696±0.057	+20	0.400±0.044	0.512±0.066	+28
AI-Feynman	0.110±0.015	0.088±0.016	+20	0.480±0.034	0.605±0.041	+26	0.300±0.038	0.405±0.057	+35
DSR	0.125±0.018	0.103±0.018	+18	0.420±0.033	0.538±0.042	+28	0.240±0.030	0.336±0.048	+40
PySR	0.085±0.012	0.066±0.012	+22	0.640±0.043	0.730±0.054	+14	0.450±0.052	0.540±0.063	+20
gplearn	0.135±0.021	0.115±0.018	+15	0.400±0.032	0.520±0.040	+30	0.220±0.026	0.319±0.041	+45
SINDy	0.155±0.024	0.136±0.021	+12	0.340±0.029	0.449±0.040	+32	0.200±0.024	0.300±0.036	+50
EQL	0.120±0.017	0.098±0.017	+18	0.460±0.033	0.561±0.041	+22	0.270±0.030	0.356±0.041	+32
GP-SR	0.130±0.020	0.109±0.016	+16	0.430±0.034	0.533±0.041	+24	0.250±0.027	0.338±0.042	+35
Symbolic PINN	0.100±0.012	0.077±0.013	+23	0.560±0.041	0.661±0.043	+18	0.380±0.050	0.475±0.046	+25
FLEXPDE-SR	0.115±0.019	0.092±0.014	+20	0.470±0.033	0.564±0.037	+20	0.290±0.040	0.377±0.037	+30
Systematic drift (time-dependent bias)									
SciMED	0.060±0.008	0.051±0.007	+15	0.720±0.054	0.792±0.065	+10	0.500±0.054	0.575±0.062	+15
AI-Feynman	0.070±0.012	0.062±0.008	+12	0.660±0.050	0.739±0.054	+12	0.440±0.055	0.519±0.064	+18
DSR	0.080±0.013	0.072±0.011	+10	0.600±0.045	0.684±0.053	+14	0.380±0.051	0.456±0.057	+20
PySR	0.058±0.010	0.050±0.007	+14	0.740±0.055	0.799±0.066	+8	0.520±0.071	0.582±0.060	+12
gplearn	0.090±0.013	0.083±0.014	+8	0.550±0.041	0.638±0.058	+16	0.320±0.037	0.400±0.054	+25
SINDy	0.100±0.012	0.093±0.013	+7	0.500±0.036	0.590±0.050	+18	0.300±0.033	0.384±0.050	+28
EQL	0.078±0.014	0.070±0.009	+10	0.610±0.037	0.683±0.045	+12	0.390±0.045	0.468±0.055	+20
GP-SR	0.085±0.013	0.077±0.014	+9	0.580±0.042	0.661±0.049	+14	0.360±0.039	0.439±0.060	+22
Symbolic PINN	0.065±0.011	0.057±0.009	+13	0.690±0.055	0.759±0.073	+10	0.470±0.052	0.540±0.057	+15
FLEXPDE-SR	0.075±0.013	0.067±0.011	+11	0.630±0.048	0.706±0.067	+12	0.410±0.058	0.484±0.064	+18

Reported values are mean ± standard deviation across all ten datasets at the maximum noise level $\sigma _n=0.05$.

### Physical consistency check

To complement fit-based metrics (MAE/$R^2$/ETD), we quantify whether discovered equations satisfy known physical constraints. For each final discovered expression *E*, we report two scalar diagnostics computed on the simulated trajectory $D_b^{(k)}$: (i) a symmetry/invariance violation score, and (ii) a boundary/initial-condition residual score. We compute $\textrm{symm}(E)$ by sampling a small set of transformations $g \sim \mathcal {G}$ that are valid for the target system (e.g., time translation for autonomous systems; Galilean boosts for applicable dynamical systems). We report the average score across experiments:

$$\textrm{Score}_{\textrm{sym}}(E) = \frac{1}{K}\sum _{k=1}^{K}\textrm{symm}^{(k)}(E).$$

Moreover, using $B(\cdot )$, we report a normalized constraint residual:

$$\textrm{Score}_{\mathrm {BC/IC}}(E) = \frac{1}{K}\sum _{k=1}^{K} \frac{\left\| B(D_b^{(k)})\right\| _2^2}{|B(D_b^{(k)})|},$$

where $|B(\cdot )|$ is the number of scalar constraint entries (nodes $\times$ times $\times$ fields).

Table 7 summarizes these results. These scores correspond to the same physical-consistency mechanisms used in the SR objective via $\textrm{symm}(E)$ and $B(\cdot )$ in Eq. (6). Across the ten benchmark systems, the dual tabular-graph representation reduces both types of violations for all SR solvers, with the largest relative gains typically observed for classical genetic-programming and sparse-regression baselines, indicating that the physics-aware graph/latent structure helps steer the search away from algebraically plausible but physically inconsistent expressions.

**Table 7:** Physical consistency check (lower is better).

SR model	Symmetry / invariance violation			BC/IC residual		
	Tab.	Dual	$\Delta (\%)$	Tab.	Dual	$\Delta (\%)$
SciMED	0.010±0.004	0.006±0.003	+40	0.014±0.006	0.008±0.004	+43
Symbolic PINN	0.012±0.005	0.007±0.004	+42	0.016±0.006	0.010±0.005	+38
FLEXPDE-SR	0.015±0.007	0.010±0.005	+33	0.018±0.008	0.012±0.006	+33
GP-SR	0.022±0.009	0.016±0.007	+27	0.025±0.010	0.018±0.008	+28
EQL	0.028±0.010	0.020±0.008	+29	0.030±0.011	0.022±0.009	+27
DSR	0.030±0.012	0.022±0.009	+27	0.034±0.013	0.026±0.011	+24
AI-Feynman	0.034±0.015	0.027±0.012	+21	0.038±0.015	0.030±0.012	+21
PySR	0.045±0.017	0.033±0.014	+27	0.050±0.018	0.036±0.015	+28
SINDy	0.060±0.020	0.040±0.016	+33	0.070±0.022	0.048±0.017	+31
gplearn	0.075±0.025	0.049±0.020	+35	0.085±0.026	0.056±0.021	+34

$\Delta (\%)$ denotes the relative reduction in violation achieved by the dual tabular-graph representation compared to the tabular baseline.

### Performance landscapes over noise and data availability

To provide an intuitive, multi-dimensional view of how robustness and data efficiency interact, we visualize the dual-representation performance over the joint grid of noise intensity $\sigma _n$ and number of experiments *K*. Each point in Fig. 7 corresponds to one $(\sigma _n, K)$ setting, and the vertical axis shows the aggregated metric value (mean across SR solvers and benchmark systems). These 3D views complement the 1D sensitivity curves (Figs. 4–5) by making it easier to identify regimes where performance degrades most strongly (high noise / low data) and how quickly it recovers as more experiments are available.
